# Efficient and Unbiased Estimation of Population Size

**DOI:** 10.1371/journal.pone.0141868

**Published:** 2015-11-04

**Authors:** Marcos Cruz, Domingo Gómez, Luis M. Cruz-Orive

**Affiliations:** Department of Mathematics, Statistics and Computer Science, Univ. de Cantabria Av. Los Castros s/n, E-39005 Santander, Spain; University of Warwick, UNITED KINGDOM

## Abstract

Population sizing from still aerial pictures is of wide applicability in ecological and social sciences. The problem is long standing because current automatic detection and counting algorithms are known to fail in most cases, and exhaustive manual counting is tedious, slow, difficult to verify and unfeasible for large populations. An alternative is to multiply population density with some reference area but, unfortunately, sampling details, handling of edge effects, etc., are seldom described. For the first time we address the problem using principles of geometric sampling. These principles are old and solid, but largely unknown outside the areas of three dimensional microscopy and stereology. Here we adapt them to estimate the size of any population of individuals lying on an essentially planar area, e.g. people, animals, trees on a savanna, etc. The proposed design is unbiased irrespective of population size, pattern, perspective artifacts, etc. The implementation is very simple—it is based on the random superimposition of coarse quadrat grids. Also, an objective error assessment is often lacking. For the latter purpose the quadrat counts are often assumed to be independent. We demonstrate that this approach can perform very poorly, and we propose (and check via Monte Carlo resampling) a new theoretical error prediction formula. As far as efficiency, counting about 50 (100) individuals in 20 quadrats, can yield relative standard errors of about 8% (5%) in typical cases. This fact effectively breaks the barrier hitherto imposed by the current lack of automatic face detection algorithms, because semiautomatic sampling and manual counting becomes an attractive option.

## Introduction

The size of a population is the total number of individual feature elements or units (e.g. organisms) constituting the population. If the latter sits in an open area, then its elements can in principle be identified and counted from aerial photographs (see for instance [1]). Often the natural reaction is to count all the elements, a task that is usually carried out ultimately by hand. In practice, however, this may be bearable for population sizes of the order of 1000 elements, although it is tedious, slow, highly-dependent on the skill of the operator and difficult to verify. It is easy to realize that proper sampling will be imperative in general. Unfortunately, proper sampling strategies are usually lacking in this context. As far as human crowds is concerned, size estimates differ widely among convention organizers, media and police [2]. Similar remarks tend to apply in ecology and other sciences. Population density is usually estimated with quadrats, but no clear criteria are given on how to place the quadrats, on how to correct the edge effects arising in quadrat counting, etc. The lack of a well defined sampling mechanism precludes not only the unbiased estimation of particle size, but also a reliable prediction of the corresponding error variance.

Automatic image analysis is not yet a reliable alternative. For instance, the automatic detection of human faces in still pictures has been studied carefully [3, 4]. As explained in [5], however, state of the art human face detectors are known to perform poorly in general due to a host of artifacts such as large pose and illumination variations, occlusions, expression variations, out-of-focus blur, and low image resolution. In the particular case of human crowds, alternative methods are arising from the recent increase in the usage of technology [6].

**Fig 1:**
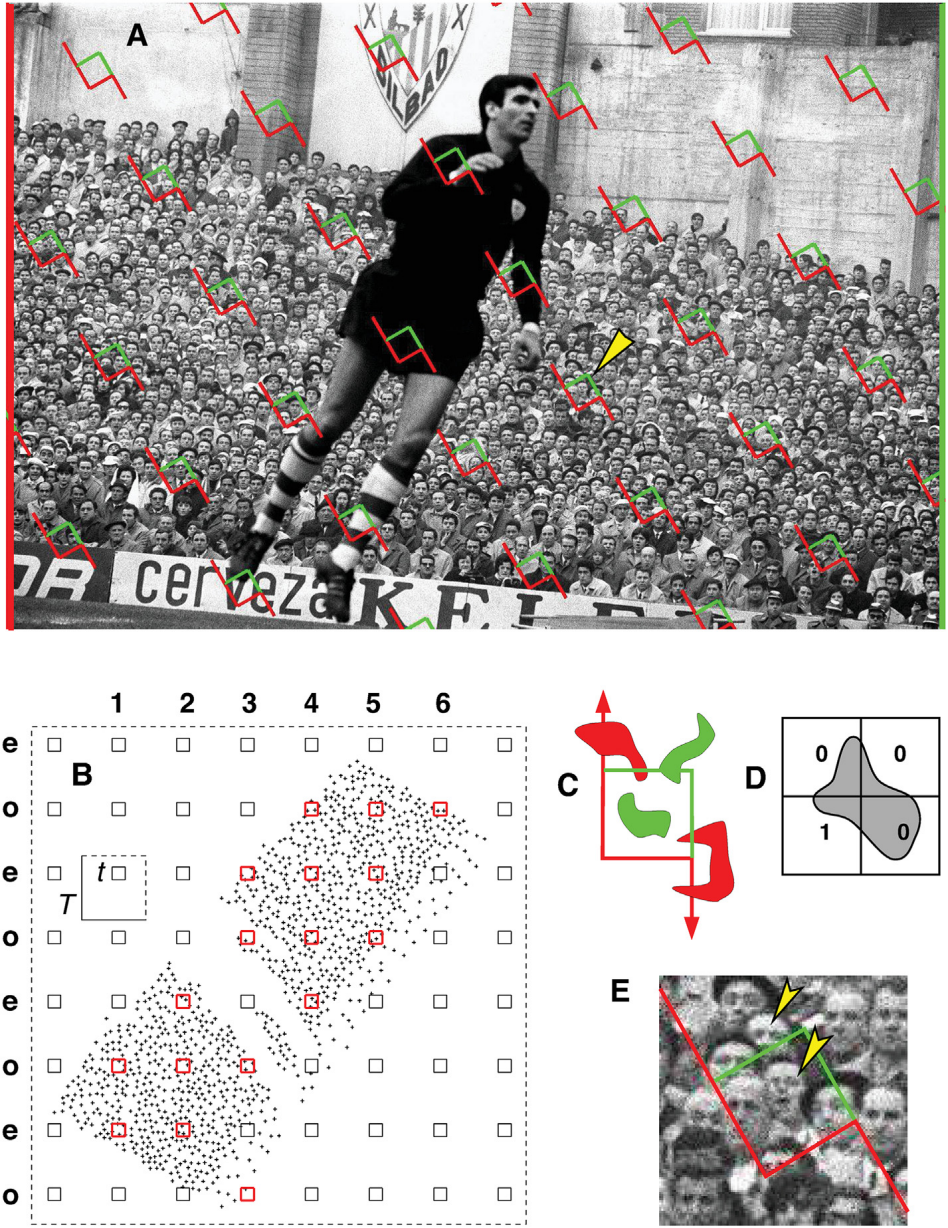
Systematic quadrat sampling and unbiased counting rule to estimate feature number in the plane. (A). Spectators in a football match (Bilbao, 1966), (original picture taken from [14]; with permission of the author). A square grid of quadrats was superimposed uniformly at random to estimate the total head number. The quadrat marked with a yellow arrowhead is magnified in (E). Size of the entire picture: 1796 × 1200 pixels. (B) Corresponding associated points—as used in our Monte Carlo automatic resampling—with the same grid superimposed on them (quadrat side length *t* = 50, fundamental box side length *T* = 250 pixels). (C) Forbidden line rule to remove edge effects in manual counting [11]. The green particles are counted in the quadrat, the red ones are not because they hit the extended forbidden edge (in red). (D) Application of the forbidden line rule to each of the four quadrats shown, yields the total correct count of one particle. (E) With the latter rule, only arrowed heads are counted.

Here we propose design based systematic sampling to estimate population size with low and predictable errors. In mathematical terms, the problem is to estimate the finite size *N* of a bounded population *Y* of particles on an observation plane. In general, a particle is defined as a compact and connected subset separated from other particles. In the human crowd context a particle is the planar projection of a human head, or a clearly distinguishable fragment of it, as observed on a photograph of a crowd, see Fig 1A. The purpose of this paper is twofold. First, to propose a design unbiased estimator $\hat{N}$ of *N*, which means that, up to practical artifacts, the mean of the error $\hat{N}-N$ over all possible samples, is zero. This is a mathematical property warranted by the sampling design, and it does not depend on population pattern and size. The only practical requirement is that the particles are unambiguously distinguishable for counting. The second purpose is to predict the error variance $\text{Var}(\hat{N})$. The later task is non trivial because the observations will be systematic, hence dependent in general. Here no variance estimator exists which is always unbiased.

The method proposed to estimate *N* is adapted from well known principles of geometric sampling for stereology, and they are widely used in many disciplines—for references see for instance [7] and [8]. Early papers on particle counting methods are [9–11]; for a review see [12].

The design unbiased estimator of *N* is described in section Design based model: Unbiased estimation of *N*. In section Variance Estimators we describe two alternative variance estimators, namely a traditional, naive one relying on independence assumptions, and a new one based on relatively recent results. Their relative performance is checked via Monte Carlo simulations on two real pictures (Fig 1A and Fig 7b of [13]) described in section Material. The simulations are facilitated by replacing each sampling unit (human head) with an associated point. This process, plus the concrete details of the simulations, the results, and their interpretation, are described in section Results and Discussion. Practical estimation procedures for arbitrary particles (as opposed to point particles) are illustrated in section Sizing a crowd in practice: numerical step-by-step procedures. Finally, section Concluding Remarks is devoted to final comments and conclusions.

## Materials and Methods

### Design based model: Unbiased estimation of *N*

The population of particles is assumed to be fixed and deterministic, and it is represented by a discrete, finite set *Y* = {*y*_1_, *y*_2_, …, *y*_*N*_}, where *y*_*i*_ denotes the *i*th particle. It is also assumed that *Y* is bounded, that is, *Y* can be contained in a disk of finite radius. To estimate the population size *N* we use systematic sampling with a uniform random (UR) test system of quadrats (also called a ‘grid of quadrats’) of a given, arbitrary orientation in the plane. Here we adopt a square grid of square quadrats. Initially, the lower left corners of the quadrats sit at the vertices of a fixed square lattice of points whose fundamental tile or box *J*_0_ is a square of side length *T* (also called the ‘lattice size’) and area *a* = *T* ^2^. The quadrats have side length *t*, (0 < *t* ≤ *T* < ∞) and area *a*′ = *t* ^2^. The UR condition is essential to the method. Strictly, a UR systematic grid Λ_*z*_ of quadrats is generated by shifting the lower left corner of a quadrat to a point *z* ∈ *J*_0_ which is UR within *J*_0_, thus dragging the whole quadrat grid together (Fig 1B). In practice, one simply throws the grid ‘at random’ over the target population *Y*. The intersection *Y* ∩ Λ_*z*_ is a systematic quadrat sample from *Y*. Let *Q* = *Q*(*Y* ∩ Λ_*z*_) denote the sample size, namely the total number of particles captured by the quadrats. Then,

$$\begin{array}{c} \hat{N}=\frac{a}{a^{\prime }}\cdot Q, \end{array}$$ (1)

is an unbiased estimator of *N*, that is, the average error of $\hat{N}$ over all the potential UR superimpositions of the grid, is zero. For completeness, a proof of the unbiasedness of $\hat{N}$ is given in S1 Appendix. The ratio *a*′/*a* ≤ 1 is the sampling fraction. Note that the estimation is direct, that is, no use is made of any quantity (such as a reference area, etc.) other than the relevant number count *Q*. By the same token, the estimator is scale independent. Another advantage of the proposed counting method is that only those particles sampled by the quadrats need to be examined, the rest may be ignored. Note also that the grid is theoretically unbounded, hence grazing quadrats hitting particles must not be ignored.

It only remains to define an unbiased counting rule to obtain *Q*, namely a rule that copes with edge effects and ensures that all the particles have identical probabilities of being sampled with a UR grid of quadrats. A convenient rule for manual counting is the forbidden line rule, see [7, 11, 12], and Fig 1C–1E. A particle is counted in a quadrat only if its has points in common with the quadrat but it does not hit the extended forbidden line of the quadrat. Alternatively, the associated point rule [7, 9, 10], is better suited for automatic particle detection, and for computer aided simulations. The rule establishes that a particle is counted in a quadrat if its associated point—namely a point attached to the particle according to a rule fixed a priori for all particles—is contained in the quadrat. Fig 1B shows all the associated points corresponding to the spectators in Fig 1A. Particle counting is now straightforward but, unfortunately, obtaining Fig 1B from Fig 1A is generally an arduous task as described in the *Material* subsection below.

Note that two different unbiased rules such as the preceding ones do not need to yield identical counts in a given quadrat—unbiasedness implies coincidence in the mean.

### Variance Estimators

We explore the performance of two alternative error variance estimators—namely the naive one based on independence, and a more elaborate one—by Monte Carlo resampling on digitized versions of Fig 1A and Fig 7b of [13]. True variances are denoted by Var(⋅), whereas variance estimators are denoted by var(⋅).

#### Estimation of the error variance of $\hat{N}$ assuming independence between quadrats

The first estimator, ${\text{var}}_{\text{ind}}(\hat{N})$, is often used [2], and it assumes independence between quadrats. Suppose that the sample *Y* ∩ Λ_*z*_ consists of *n* ≥ 2 non empty systematic quadrats capturing {*q*_1_, *q*_2_, …, *q*_*n*_} particles, respectively. Then *q*_1_ + *q*_2_ + … + *q*_*n*_ = *Q* is the total number of sampled particles. Further let var(*q*_1_) denote the sample variance of the {*q*_*i*_}. Then,

$$\begin{array}{c} {\text{var}}_{\text{ind}}\left(\hat{N}\right)={\left(\frac{a}{a^{\prime }}\right)}^{2}\cdot n\cdot \text{var}\left(q_{1}\right). \end{array}$$ (2)

#### Estimation of the error variance of $\hat{N}$ using the Cavalieri slices design

The second error variance predictor, ${\text{var}}_{\text{Cav}}(\hat{N})$, contemplates quadrat dependence, and it is based on G. Matheron’s transitive theory [15]. In [16] the target parameter was volume, and the variance estimator was derived for a volume estimator obtained from Cavalieri slices produced by parallel systematic slabs normal to an arbitrary sampling axis. In our context the slabs are planar stripes of thickness *t* > 0 a constant distance *T* > *t* apart (namely the distance between left hand side stripe edges say), with a UR positon of the left hand side edge in the interval [0, *T*). The estimator was extended to the case in which the target parameter is particle number in [17–19]. Here we adopt a suitable combination of these methods. The idea is to regard the quadrat sample as a two stage sample. The first stage involves Cavalieri stripes, and in the second, each stripe is subsampled in turn by a perpendicular series of Cavalieri stripes with the same parameters *t*, *T*. The result is clearly equivalent to a grid of systematic quadrats with the latter parameters. Here, the required notation is different from the other sections. Define

- *τ* = *t*/*T* ∈ (0, 1], stripe sampling fraction.
- *n*: number of stripes encompassing the particle population, (*n* > 2).
- *n*_*i*_: number of quadrats subsampled within the *i*th stripe, *i* = 1, 2, …, *n*.
- *q*_*ij*_: number of particles captured by the *j*th quadrat within the *i*th stripe, *j* = 1, 2, …, *n*_*i*_.
- *Q*_*oi*_, *Q*_*ei*_: total numbers of particles captured by the odd numbered, and by the even numbered quadrats, respectively, within the *i*th stripe.
- $Q_{i}=\sum_{j=1}^{n_{i}}q_{ij}$, total number of particles sampled in the *i*th stripe. Note that *Q*_*i*_ = *Q*_*oi*_ + *Q*_*ei*_.
- $Q=\sum_{i=1}^{n}Q_{i}$, total number of sampled particles.

Now, the estimator given by Eq 1 may be written $\hat{N}=\tau^{-2}\cdot Q$. The following estimator of $\text{Var}(\hat{N})$ is obtained from Eq (3.3) of [18] with *q* = 0, namely,

$$\begin{array}{ccc} {\text{var}}_{\text{Cav}}\left(\hat{N}\right) & = & \frac{1}{6}\cdot \frac{{\left(1-\tau \right)}^{2}}{\tau^{4}\left(2-\tau \right)}\cdot \left[3\left(C_{0}-\nu_{n}\right)-4C_{1}+C_{2}\right]+\frac{\nu_{n}}{\tau^{4}}, \end{array}$$ (3)

$$\begin{array}{ccc} C_{k} & = & \sum_{j=1}^{n-k}Q_{j}Q_{j+k},\;\;k=0,1,2. \end{array}$$ (4)

The first term in the right hand side of Eq (3) estimates the between stripes variance contribution, whereas *τ* ^−4^ *ν*_*n*_ estimates the within stripes contribution (namely the contribution of the variation between quadrats within stripes). The latter contribution may be estimated using the splitting estimator given in [17]. The relevant within stripes variance term is obtained from Eq (4.1) of the latter paper (with ${\hat{\nu }}_{2}=0$), namely:

$$\begin{array}{c} \nu_{n}=\frac{{\left(1-\tau \right)}^{2}}{3-2\tau }\cdot \sum_{i=1}^{n}{\left(Q_{oi}-Q_{ei}\right)}^{2}. \end{array}$$ (5)

*Remarks:* Let *N*_*i*_ denote the total, true number of particles captured by the *i*th entire stripe (this notation is different from that used in other sections). Then the Cavalieri stripes estimator of *N*, namely *τ* ^−1^(*N*_1_ + *N*_2_ + … + *N*_*n*_), is unbiased, and its variance is estimated by the first term in the right hand side of Eq (3). Further, an unbiased estimator of *N*_*i*_ is ${\hat{N}}_{i}=\tau^{-1}Q_{i}$. The random error $e_{i}={\hat{N}}_{i}-N_{i}$ is assumed to have zero mean and variance $\sigma_{i}^{2}\geq 0$. In the derivation of Eq (5), the within stripe errors {*e*_1_, *e*_2_, …, *e*_*n*_} are assumed to be independent between stripes, whereby the right hand side of Eq (5) estimates the quantity $\tau^{2}\cdot (\sigma_{1}^{2}+\sigma_{2}^{2}+\ldots +\sigma_{n}^{2})$.

### Material

To check our model and estimators we chose the crowd pictures shown in Fig 1A and Fig 7b of [13]. To facilitate programming each particle was replaced with its associated point which was approximately the centre of the smallest rectangle enclosing a sampling unit (i.e. a head or head fragment). For Fig 1A, this task was performed with the aid of the OpenCV software (Open Source Computer Vision), with tedious additional manual editing. We chose Fig 1A since most of the visible faces are frontal, and well resolved, hence OpenCV was able to detect about 80% of the faces, including some false detections. However, this is generally not the case, hence the failure rate for OpenCV may be expected to be higher in most real crowd images. Units hitting the left hand side border of the picture were discarded, the ones hitting the right hand side border were retained; this rule would eliminate double counting in potentially adjacent pictures.

For Fig 7b of [13] the associated point coordinates were borrowed from the dataset mentioned in [13].

Fig 1A and Fig. 7b of [13] contain *N* = 1120 (Fig 1B) and *N* = 4633 associated points, respectively.

In practice a picture such as Fig 1A or Fig 7b of [13], does not need to constitute a population of interest in itself, but rather a sample from a high-resolution panoramic image. For our present purposes, however, each of the two pictures is regarded as a target population in itself.

## Results and Discussion

### Empirical assessment of the variance estimators by Monte Carlo resampling with systematic quadrats on real pictures

**Fig 2:**
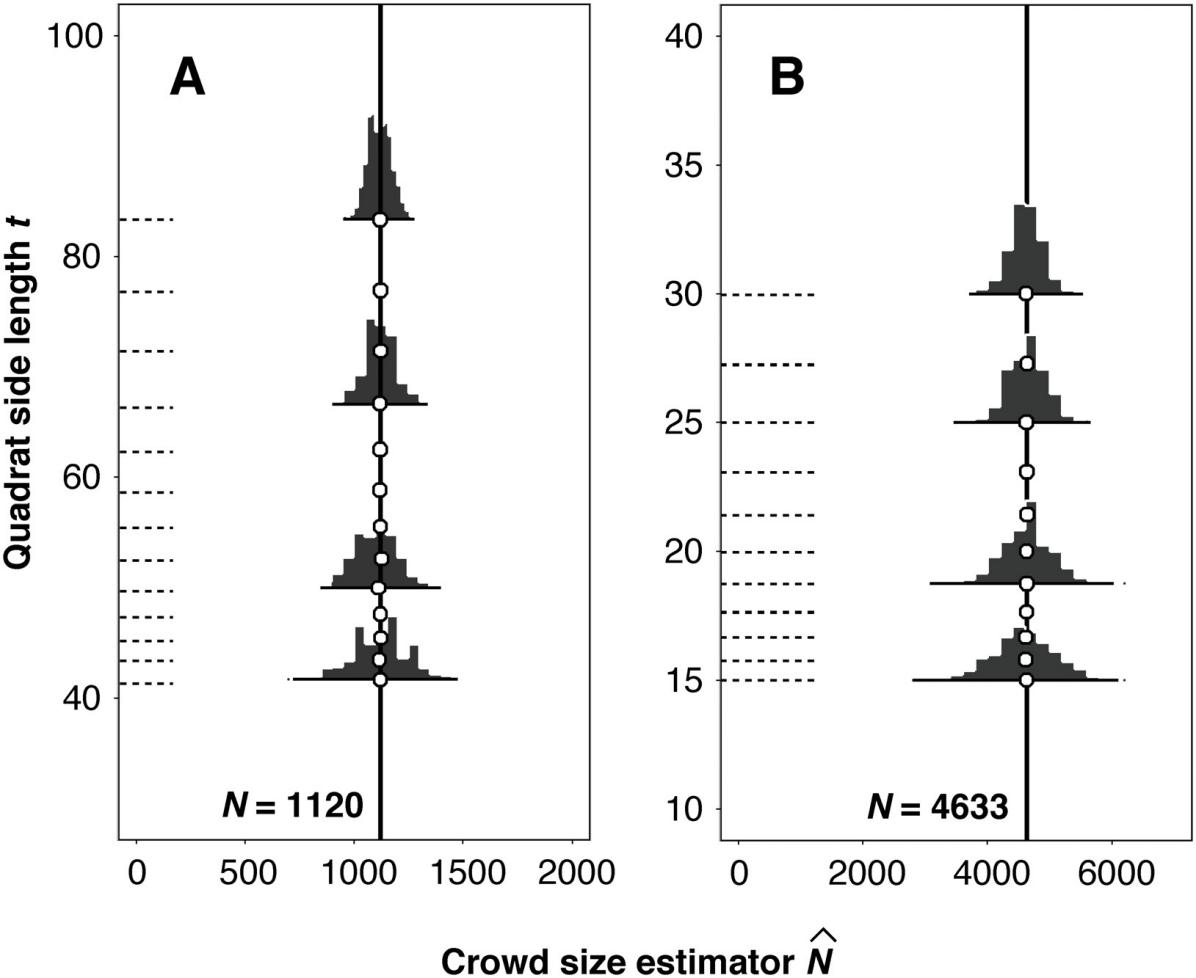
Empirical, Monte Carlo histograms of crowd number estimates. (A) Empirical distributions of the number estimator given by Eq (1), obtained for Fig 1A from 1024 Monte Carlo superimpositions of quadrat grids of different sizes. The side length of the fundamental box was *T* = 250 pixels. As expected, the histogram means always coincide with the true crowd size because the estimator is unbiased. (B) Analogous data for Fig 7b of [13]. Here *T* = 150 pixels.

**Fig 3:**
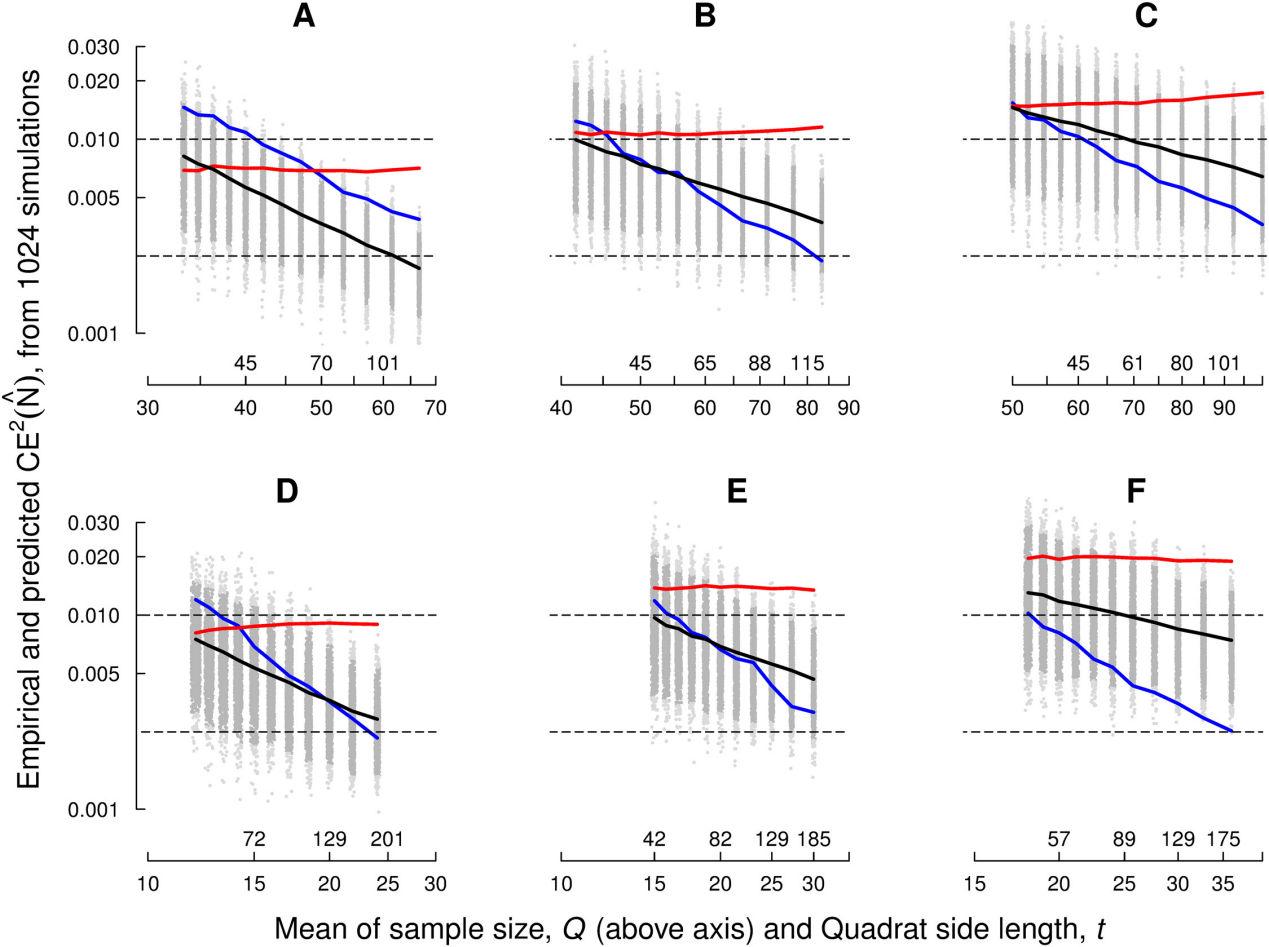
Monte Carlo empirical square coefficient of error and corresponding mean predictors. (A, B, C) Monte Carlo results corresponding to Fig 1A, for fundamental box side lengths *T* = 200,250,300 pixels, respectively, and for different quadrat side lengths in each case. The equivalent mean sample sizes are also shown. The empirical square coefficient of error (= error variance divided by *N* ^2^) is represented in blue, whereas the corresponding mean predictors obtained with Eqs (2) and (3), are represented in red and black colour, respectively. Grey dots represent all the replicated values of ${\text{ce}}_{\text{Cav}}^{2}(\hat{N})$. The dark grey dots lie between the 2.5% and 97.5% quantiles. The broken horizontal lines correspond to 5% and 10% coefficients of error, respectively. (D, E, F) Analogous data for Fig 7b of [13]—here *T* = 120,150,180 pixels, respectively.

The empirical distribution of $\hat{N}$ and the performances of ${\text{var}}_{\text{ind}}(\hat{N})$ and ${\text{var}}_{\text{Cav}}(\hat{N})$ were checked by Monte Carlo resampling on Fig 1A and Fig 7b of [13] for the values of *t* and *T* considered in Fig 2 and Fig 3. To increase precision [20], each picture was tilted 60° prior to sampling in order to avoid parallelism between the sampled stripes and the edges of the picture, see Fig 1B. For each pair (*t*, *T*) a total of *K* ^2^ = 32^2^ = 1024 replicated superimpositions of the grid Λ_*z*_ onto *Y* were generated, corresponding to *K* ^2^ random replications of the point *z* within *J*_0_. Instead of generating independent replications, we adopted a systematic design, which should be expected to be more efficient in most cases. Thus, a UR square subgrid of *K* × *K* points of coordinates {(*x*_*i*_, *y*_*j*_, *i*, *j* = 1, 2, …, *K*)} was generated within *J*_0_ with a gap Δ = *T*/*K* between points, namely,

$$\begin{array}{c} \left\{\left(x_{i}=\left(U_{1}+i-1\right)\Delta ,y_{j}=\left(U_{2}+j-1\right)\Delta \right)\right\} \end{array}$$ (6)

where *U*_1_, *U*_2_ are independente UR numbers in the interval [0, 1). Therefore, the whole set of *K* ^2^ replications required a pair of random numbers only. For each pair (*U*_1_, *U*_2_), relabel the *K* ^2^ subgrid points as {*z*_*k*_, *k* = 1, 2, …, *K* ^2^}. For each *k*, the corresponding sample total,

$$\begin{array}{c} Q_{k}=Q\left(Y\cap \Lambda_{z_{k}}\right), \end{array}$$ (7)

was computed automatically with the aid of a simple point-in-polygon algorithm (http://www.ariel.com.au/a/python-point-int-poly.html). A particular superimposition *Y* ∩ Λ_*z*_*k*__ is illustrated in Fig 1B. The corresponding design unbiased estimator of the population size *N* was,

$$\begin{array}{c} {\hat{N}}_{k}={\left(T/t\right)}^{2}\cdot Q_{k}. \end{array}$$ (8)

The empirical mean and variance of $\hat{N}$ were computed respectively as follows,

$$\begin{array}{ccc} E_{e}\left(\hat{N}\right) & = & {\left(T/t\right)}^{2}\cdot K^{-2}\sum_{k=1}^{K^{2}}Q_{k}, \end{array}$$ (9)

$$\begin{array}{ccc} {\text{Var}}_{e}\left(\hat{N}\right) & = & K^{-2}\sum_{k=1}^{K^{2}}{\left\{{\hat{N}}_{k}-E_{e}\left(\hat{N}\right)\right\}}^{2}. \end{array}$$ (10)

These values are supposed to be very close to their respective true values. Empirical distributions of the $\left\{{\hat{N}}_{k}\right\}$ are displayed in Fig 2, confirming unbiasedness and a moderate dispersion.

We also computed the corresponding *K* ^2^ replicates $\left\{{\text{var}}_{\text{ind}}({\hat{N}}_{k})\right\}$, $\left\{{\text{var}}_{\text{Cav}}({\hat{N}}_{k})\right\}$. The empirical square coefficient of errors:

$$\begin{array}{ccc} {\text{ce}}_{\text{ind}}^{2}\left(\hat{N}\right) & = & \frac{1}{N^{2}K^{2}}\sum_{k=1}^{K^{2}}{\text{var}}_{\text{ind}}\left(\hat{N_{k}}\right) \end{array}$$ (11)

$$\begin{array}{ccc} {\text{ce}}_{\text{Cav}}^{2}\left(\hat{N}\right) & = & \frac{1}{N^{2}K^{2}}\sum_{k=1}^{K^{2}}{\text{var}}_{\text{Cav}}\left(\hat{N_{k}}\right) \end{array}$$ (12)

are compared with the corresponding empirical (’true’) value,

$$\begin{array}{c} {\text{ce}}_{e}^{2}\left(\hat{N}\right)={\text{Var}}_{e}\left(\hat{N}\right)/N^{2}, \end{array}$$

in Fig 3.

**Fig 4:**
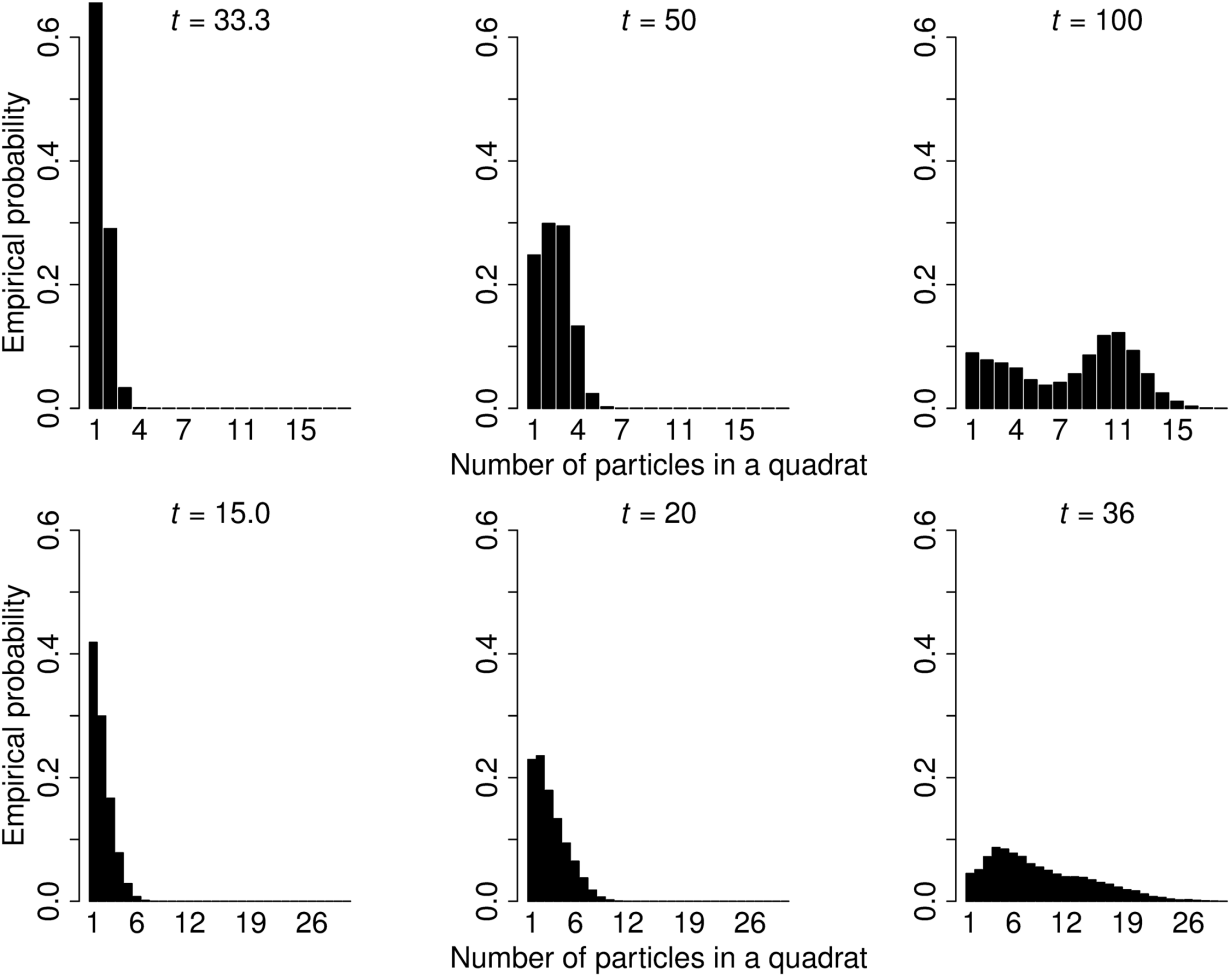
Empirical probabilities of particle number per non-empty quadrat. Empirical probabilities of the number of particles in non-empty quadrats in Fig 1A (top row) and Fig 7b of [13] (bottom row). Quadrat sizes *t* = 33.3,50,100 pixels and *t* = 15,20,36 pixels are considered for Fig 1A and Fig 7b of [13], respectively.

The estimator var_ind_, showed a poor performance. This is not unexpected since the indpendence assumption clearly fails. In addition it looks a bit paradoxical that ${\text{ce}}_{\text{ind}}^{2}$ does not decrease as *t* and *Q* increase. This can be explained from the following two facts: first, the coefficient of variation among quadrat contents increases with quadrat size *t*, and second, the number of non empty quadrats increases relatively slowly as *t* increases for each value of *T*. The corresponding graphs are not shown—instead, the empirical distribution of the non empty quadrat contents is displayed in Fig 4 for different quadrat sizes. The contents of large quadrats tends to exhibit a bimodal distribution approaching an unfavourable ‘U’ shape which causes the coefficient of variation to increase abnormally. The bimodality is probably due to perspective effects and to grazing quadrats contributing low quadrat counts. In addition, Fig 1A roughly consists of two different subpopulations: spectators sitting in the front ranks are more sparse than the remaining, standing spectators.

The results for ${\text{ce}}_{\text{Cav}}^{2}$ look more encouraging, but still subject to improvement.

## Sizing a crowd in practice: numerical step-by-step procedures

### Example of Fig 1B

The parameters of the grid are *t* = 50, *T* = 250, hence *τ* = *t*/*T* = 0.2. The non empty quadrats in Fig 1B are contained in the 6 vertical stripes numbered {1, 2, …, 6}. The corresponding particle counts in the individual quadrats (from bottom to top in the figure) are displayed in Table 1.

**Table 1:** Individual quadrat counts and preliminary calculations corresponding to Fig 1B.

	1	2	3	4	5	6	Total
o	0	0	1	0	0	0	
e	5	3	0	0	0	0	
o	4	3	3	0	0	0	
e	0	2	0	4	0	0	
o	0	0	2	4	4	0	
e	0	0	3	3	3	0	
o	0	0	0	2	2	2	
*Q*_*oi*_	4	3	6	6	6	2	
*Q*_*ei*_	5	5	3	7	3	0	
(*Q*_*oi*_−*Q*_*ei*_)^2^	1	4	9	1	9	4	28
*Q*_*i*_	9	8	9	13	9	2	50

Individual quadrat counts and preliminary calculations corresponding to Fig 1B. ‘o’ and ‘e’ refer to odd and even numbered quadrats, respectively, within the slices.

The estimate of *N* is

$$\begin{array}{c} \hat{N}={\left(\frac{T}{t}\right)}^{2}Q=25\cdot 50=1250. \end{array}$$ (13)

Recall that the true vale was *N* = 1120. Now, Eq (5) yields,

$$\begin{array}{c} \nu_{6}=\frac{{\left(1-0.2\right)}^{2}}{3-2\cdot 0.2}\cdot 28=6.892308. \end{array}$$ (14)

Further, Eq (3) yields,

$$\begin{array}{ccc} {\text{var}}_{\text{Cav}}\left(\hat{N}\right) & = & \frac{1}{6}\cdot \frac{{\left(1-0.2\right)}^{2}}{0.2^{4}\left(2-0.2\right)}\left[3\left(480-6.892308\right)-4\cdot 396+292\right]\\ & & +\frac{6.892308}{0.2^{4}}\\ & = & 4715.669+4307.692\\ & = & 9023.361. \end{array}$$ (15)

Thus, the estimate of the percent coefficient of error (or relative standard error) of the number estimator is,

$$\begin{array}{c} {\text{ce}}_{\text{Cav}}\left(\hat{N}\right)=100\cdot \frac{\sqrt{9203.361}}{1250}\approx 7.60\% \end{array}$$ (16)

which is a reasonable precision taking into account that only 50 particles were counted. The between and within slices contributions are $100\sqrt{4715.669}/1250\approx 5.49\%$ and $100\sqrt{4307.692}/1250\approx 5.25\%$, respectively.

On the other hand, the naive variance estimate assuming independence between quadrats yields, according to Eq (2),

$$\begin{array}{c} {\text{var}}_{\text{ind}}\left(\hat{N}\right)={\left(\frac{250}{50}\right)}^{4}\cdot 17\cdot \text{var}\left({\hat{q}}_{1}\right)=11250, \end{array}$$ (17)

which corresponds to a coefficient of error of $100\sqrt{11250}/1250\approx 8.49\%$.

The 1024 Monte Carlo samples yielded an empirical, nearly true variance ${\text{Var}}_{e}(\hat{N})=9839.971$. Thus, the estimate ${\text{var}}_{\text{Cav}}(\hat{N})=9023.361$ obtained above was fairly satisfactory. On the other hand, the Monte Carlo mean of ${\text{var}}_{\text{ind}}(\hat{N})$ was 19129.27, namely a gross overestimate, as illustrated in Fig 3B.

### Planning a population sizing design from the outset

Practical criteria to design a grid which is efficient and convenient to use are:

1. Aim at a total count *Q* of between 50 and 150 particles, according to whether the pattern of the particles is judged to be fairly homogeneous (i.e. the population density is seen to vary little in different regions of the picture), or relatively heterogeneous.
2. Aim at counting no more that 4 or 5 particles per quadrat.

The preceding criteria imply that the planned number of nonempty quadrats may lie between 20 and 50.

It is worth emphasizing that no ‘guess’ or pilot estimate of the target size is required to plan the sampling design. Furthermore, the method will work for any population size.

**Fig 5:**
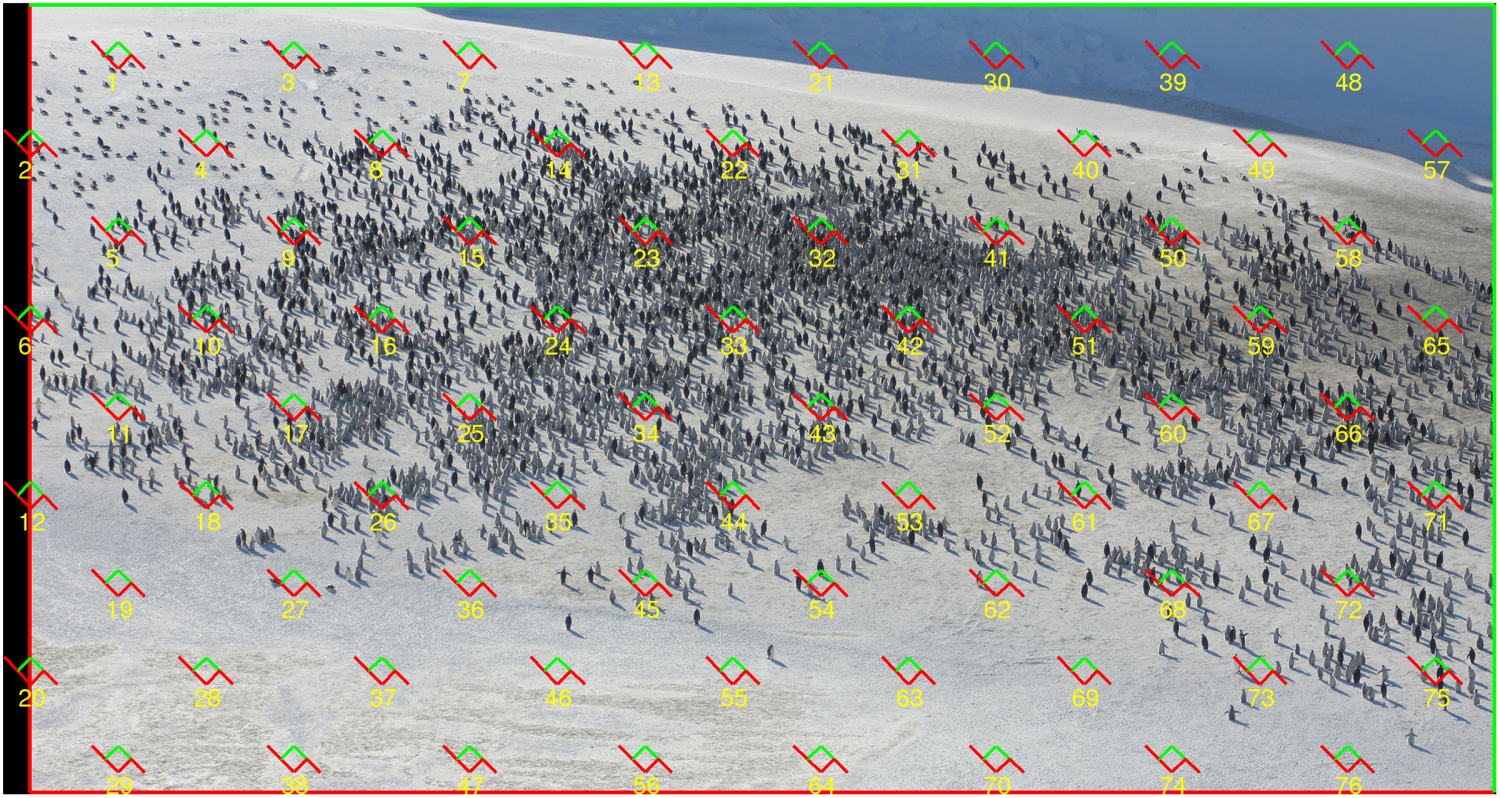
A practical protocol for systematic quadrat sampling. Aerial view of an emperor penguin colony on November 2nd, 2012. Photograph by Robin Cristofari, from an altitude of ca. 1,000 feet, Fig 3 of [1]. Picture size: 2359 × 2808 pixels. The adopted quadrat grid with *T* = 200, *t* = 30 pixels was superimposed uniformly at random with a tilting of 45° to reduce error variance, see text.

**Fig 6:**
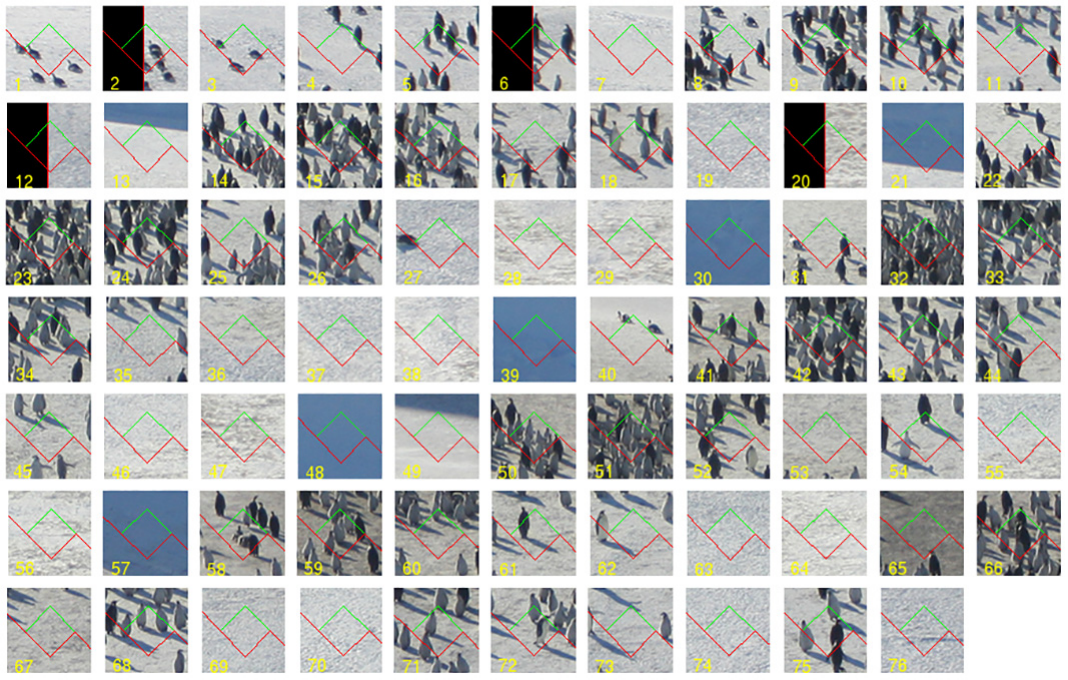
Quadrats used to estimate the total number of people in Fig 5. Magnified version of the 50 quadrats marked in Fig 5.

As an example consider Fig 5. The size of the relevant part of the picture, namely of the region containing the penguins, was approximately 2359 × 826 pixels. The inhomogeneity of the population pattern suggests to aim at counting *Q* = 150 in about 50 quadrats. To obtain a total of 50 quadrats, we require a fundamental box side length of $T=\sqrt{2359\cdot 826/50}\approx 197$ pixels. We adopted *T* = 200 pixels. Further, since we aim at counting *Q* = 150 penguins, the mean number of counts per quadrat will be about 3, which sounds reasonable. To capture 1 − 6 penguins in a quadrat, inspection of the picture suggests a quadrat side length of *t* = 30 pixels. Random superimposition of the grid at 45° tilting yielded the following 76 quadrat counts:

$$\begin{array}{c} \{\{0,1\},\{0,1,3,0\},\{0,1,5,3,1,0\},\{0,5,9,5,0,2,0,0\},\{0,0,6,5,0,4,0,0,0\},\\ \{0,0,8,7,4,0,0,0,0\},\{0,2,2,6,4,1,0,0,0\},\{0,0,5,9,1,0,1,0,0\},\\ \left\{0,5,5,1,1,0,0,0\},\{0,3,0,2,0,0\},\{2,2,0,0\},\{1,0\}\right\} \end{array}$$ (18)

corresponding to the quadrats shown in Fig 6. The 12 subsets within curly brackets correspond to the 12 tilted stripes or ‘slices’ seen in Fig 5. The total count was *Q* = 123 and (*T*/*t*)^2^ = 400/9, whereby $\hat{N}=5467$ penguins. Application of Eq (3) yielded ${\text{ce}}_{\text{Cav}}(\hat{N})=5.08\%$. Counting was fairly cursory (without too much attention) and took only a few minutes. Note that the error variance is reasonably low, despite the inhomegenous distribution of the penguin population. A factor contributing to the low error variance in this case was the smoothness of the total slice counts, {1, 4, 10, 21, 15, 19, 15, 16, 12, 5, 4, 1}, owing in part to the fact that the test grid was conveniently tilted 45°. Theory shows that an important component of the variance trend is the sum of squares of the jumps of the measurement function (of which the preceding sequence is a systematic sample), see [21, 22]. Thus, aiming at a smooth dome shape of such sequence is important [23].

## Concluding Remarks

As stated in the Introduction, apart from the basic requirement that all the particles in the population should be unambiguously identifiable for counting, there are no other practical limitations to the method. If the sampling protocol is respected, then the resulting estimate is necessarily design unbiased in all cases, irrespective of population pattern and size. Unbiasedness means that the mean of all potential estimates $\hat{N}$ obtained by repeated sampling will coincide with the true population size *N*. This is a truth founded on the mathematics of the sampling design (see S1 Appendix), and it cannot be verified by experiment unless the true value of *N* is known, (see e.g. Fig 2).

Our conclusions are the following. (a) For the first time we have implemented a direct, unbiased and efficient design to estimate population size in still pictures, provided that every sampling unit is distinguishable for counting. (b) After sampling, manual counting is a realistic option because sample sizes from 50 to 120 will usually yield unbiased number estimates with a moderate relative standard error, irrespective of population pattern and size. This fact effectively breaks the barrier hitherto imposed by the unavailability of automatic face detection in general. (c) The quadrat grid parameters can be easily designed in each case combining the preceding values with the convenience criterion that the number of individuals counted per quadrat should vary from 1 to 5. (d) A new error variance prediction formula has also been developed and Monte Carlo checked to have a reasonable performance. This probably owes to the fact that the formula takes the correlation structure of the data into account.

Naturally the present method can in principle be used to count people, animals, or any kind of distinguishable objects.

## Supporting Information

S1 AppendixProof of unbiasedness of $\hat{N}$.(PDF)
