# Supplementary material for: Efficient and Unbiased Estimation of Population Size
Source: PLoS One. 2015 Nov 4;10(11):e0141868. doi: 10.1371/journal.pone.0141868 (PMC4633052; doi:10.1371/journal.pone.0141868)
Supplement: S1 Appendix — (PDF) [file pone.0141868.s001.pdf]

Efficient and unbiased estimation of population size: Supporting Information

Marcos Cruz *, Domingo Gómez, Luis M. Cruz-Orive

Department of Mathematics, Statistics and Computer Science, Univ. de Cantabria
Av. Los Castros s/n, E-39005 Santander, Spain

* marcos.cruz@unican.es

S1 Appendix

Proof of unbiasedness of $\widehat{N}$

First, choose a non void fundamental tile, or fundamental box $J_0 \subset \mathbb{R}^2$ which can generate a partition of the plane (namely a paving without overlapping), that is, there exists a enumerable set of translations $\{\tau_k, k \in \mathbb{Z}\}$ such that

$$\bigcup_{k\in\mathbb{Z}} J_{\tau_k} = \mathbb{R}^2, J_{\tau_k} = J_0 + \tau_k, J_{\tau_k} \cap J_{\tau_l} = \quad \text{if} \quad k \neq l, \quad k, l \in \mathbb{Z}. \tag{S1.1}$$

Thus, the tiles $J_k, k \in \mathbb{Z}$ are congruent and they constitute a partition of the plane. Every tile J_{τ_k} can be brought to coincide with the fundamental tile J_0 by means of a translation $-\tau_k$ which leaves the partition unchanged. Examples of fundamental tiles are parallelograms, regular hexagons, etc., - here the square shape was adopted. Next, choose a basic sampling probe, or quadrat $T_0 \subset J_0$. For convenience suppose that the associated points of J_0 and T_0 sit at the origin. In principle no restriction needs to be imposed on the shape of T_0. It is advisable, however, that T_0 and J_0 have the same shape, so that, as the size of T_0 is increased continuously, T_0 can coincide with J_0 and strong consistency is thereby achieved [1]. Now, choose a UR point $z \in J_0$ namely a point with a constant probability density in the fundamental tile,

$$f(z) = \frac{1_{J_0}(z)}{A(J_0)}, \tag{S1.2}$$

where $1_A(x)$ denotes the indicator function of a set A, namely $1_A(x) = 1$ if $x \in A$ and $1_A(x) = 0$ if $x \notin A$. The translate of the quadrat T_0 by the vector z is written $T_z = T_0 + z$. Then, the following enumerable set of quadrat translates,

$$\Lambda_z = \{T_{z+\tau_k}, \quad k \in \mathbb{Z}\}, \quad z \sim \mathrm{UR}(J_0), \tag{S1.3}$$

represents a UR grid of quadrats of fixed orientation in the plane. Superimpose the UR grid Λ_z on the particle population Y, and let $Q(Y \cap \Lambda_z)$ denote the total number of particles sampled by the quadrats. Then,

$$\widehat{N} = \frac{A(J_0)}{A(T_0)} \cdot Q(Y \cap \Lambda_z), \tag{S1.4}$$

is an UE of N, that is $\mathbb{E}(\widehat{N}) = N$. Note that $A(T_0)/A(J_0)$ is the sampling fraction.

The following proof assumes, without loss of generality, that the particles are sampled with the associated point rule. Equivalently, the population consists of N point particles, i.e., $Y = \{y_1, y_2, \ldots, y_N\}$. Consider first the integral of $Q(y \cap T_z)$ for a single point particle $y \in \mathbb{R}^2$ and a bounded mobile probe T_z, $z \in \mathbb{R}^2$ of a fixed orientation, equipped with the motion invariant density $\mathrm{d}z = \mathrm{d}z_1\mathrm{d}z_2$ in the plane [2]. Then,

$$\begin{aligned} \int_{\mathbb{R}^2} Q(y \cap T_z)\mathrm{d}z &= \int_{\mathbb{R}^2} 1_{T_z}(y)\mathrm{d}z \\ &= \int_{\mathbb{R}^2} 1_{T_0}(y+z)\mathrm{d}z \\ &= A(T_0). \end{aligned} \tag{S1.5}$$

For the whole population Y of N point particles, the corresponding integral reads,

$$\begin{aligned} \int_{\mathbb{R}^2} Q(Y \cap T_z)\mathrm{d}z &= \int_{\mathbb{R}^2} Q\left(\cup_{i=1}^N y_i \cap T_z\right)\mathrm{d}z \\ &= \sum_{i=1}^N \int_{\mathbb{R}^2} Q(y_i \cap T_z)\mathrm{d}z \\ &= N \cdot A(T_0). \end{aligned} \tag{S1.6}$$

Next we decompose the preceding integral into a sum of integrals extended over the tiles of the aforementioned partition, namely,

$$\begin{aligned} N \cdot A(T_0) &= \int_{\mathbb{R}^2} Q(Y \cap T_z)\mathrm{d}z \\ &= \sum_{k\in\mathbb{Z}} \int_{J_{\tau_k}} Q(Y \cap T_z)\mathrm{d}z \\ &= \sum_{k\in\mathbb{Z}} \int_{J_0} Q(Y \cap T_{z+\tau_k})\mathrm{d}z \\ &= A(J_0) \int_{J_0} Q(Y \cap \Lambda_z)\frac{\mathrm{d}z}{A(J_0)} \\ &= A(J_0) \cdot \mathbb{E}\left\{Q(Y \cap \Lambda_z)\right\}, \end{aligned} \tag{S1.7}$$

where the expectation is with respect to the UR probability element of the point $z \in J_0$, see Eq.(S1.2), and $Q(Y \cap \Lambda_z)$ is defined as above. Thus $\mathbb{E}\widehat{N} = N$, as claimed.

The foregoing technique is a direct application of a theorem given in Ch. 8 of [2] - see also Eq. 4.6 in [3].

References

1. Gual-Arnau X, Cruz-Orive LM. Consistency in systematic sampling. Adv Appl Probab. 1996;28(4):982–992.

2. Santaló LA. Integral Geometry and Geometric Probability. Addison-Wesley, Reading, Massachusetts; 1976.

3. Cruz-Orive LM. Stereology: meeting point of integral geometry, probability, and statistics. Math Notae. 2001;41:49–98.
